# An audit of waiting times in the outpatient clinic in Inverness Sector A NHS Highland during the COVID-19 pandemic

**DOI:** 10.1192/bjo.2021.331

**Published:** 2021-06-18

**Authors:** Oksana Zinchenko, Jennifer Hyland

**Affiliations:** 1 New Craigs Hospital, NHS Highland, NHS Education for Scotland; 2 New Craigs Hospital, NHS Highland

## Abstract

**Aims:**

This audit was to assess and improve the organizational efficiency of referrals to Inverness Sector A Outpatient Service. The referrals were audited to measure the average waiting time from referral to first offered outpatient appointment and to assess the proportion of patients waiting longer than 12 weeks.

**Method:**

The audit included routine referrals to the CMHT Inverness Sector A, NHS Highland from GP practices: Kingsmills, Burnfield, Riverside, Fairfield, Foyers and Drumnadrochit Medical Practices. The number of referrals and the number and proportion of clients given appointments for assessments were calculated. Referrals were received directly from primary care and the Mental Health Liaison Team or following Out of Hours contacts at the Mental Health Assessment Team.

Data were collected retrospectively: referrals from 1 Jan 2020–31 Aug 2020. Sample size came to 160 patients aged 16–65 years. Data were collected via review of recorded documentation on the NHSH electronic patient record systems (SCIstore), from 5th–25th January 2021.

**Result:**

160 patients (male 82, female 78) were referred from 1 Jan to 1 Sept 2020. Of these, 140 (87.5%) were given an appointment for an assessment. The mean waiting time was 12 weeks for 103 patients (64%), with 57 patients (36%) waiting longer than 13 weeks. The bimodal distribution of waiting times prompted an analysis of those with longer waiting times. In some instances, appointments were delayed because patients either did not attend (DNA) or cancelled their appointments. Reasons for delays included: postponement until further information was available; cancellation of meetings or patients DNA. In 20 cases (12.5%), the referrals deemed inadequate, prompting further liaison with the referrer for clarification about the nature of the problem and previous psychological interventions.

**Conclusion:**

The number of transactions (any amendment to a patient record) was higher than the number of patients affected, as several transactions can relate to one patients’ record.

Most referrals are vetted in advance via the daily Inverness triage huddle. Ways of improving the quality of information provided by referrers would be explored.

On receipt of each referral, the date of the 12 week deadline would be calculated and highlighted in a database.

The cross-sector (Highland wide) standardisation will add clarity about medical capacity, that does not involve use of excessive clinician time.

